# New evidence linking lifestyle factors to blood pressure: Focus on 2024 findings

**DOI:** 10.1038/s41440-026-02549-0

**Published:** 2026-02-05

**Authors:** Chisa Matsumoto

**Affiliations:** 1 Center for Health Surveillance & Preventive Medicine, Tokyo Medical University Hospital, Tokyo, Japan; 2 Department of Cardiology, Tokyo Medical University, Tokyo, Japan; 3 University of Pennsylvania, Perelman School of Medicine, Division of Cardiovascular Medicine, Philadelphia, USA

**Keywords:** Nutrition, salt, sleep, SAS, lifestyle

## Abstract

Lifestyle is closely linked to blood pressure (BP), making lifestyle modification essential for BP management. In 2024, numerous intriguing studies were reported on lifestyle factors and BP. This mini review summarizes notable research on lifestyle factors and BP published in Hypertension Research and other leading journals from 2024 through the first half of 2025, with particular focus on sleep, air pollution, and dietary factors (mainly sodium and potassium).

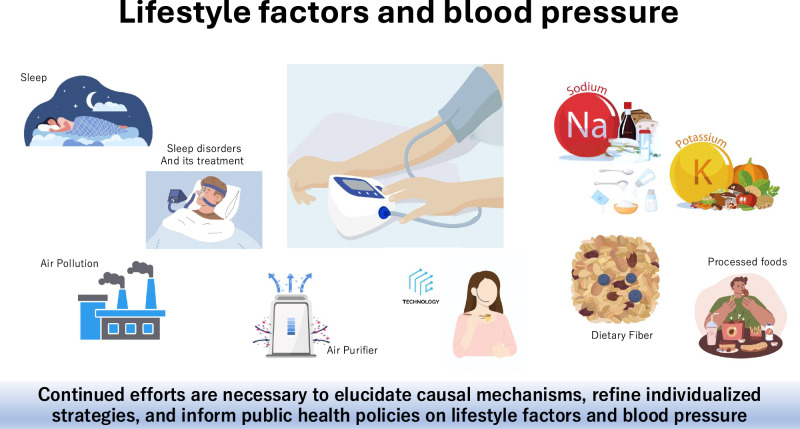

## Sleep and blood pressure

The American Heart Association’s Life’s Essential 8 includes eight key lifestyle components for cardiovascular health, with “sleep” newly added in 2022 to the earlier Life’s Simple 7 [1]. Sleep apnea syndrome (SAS), one of the most common sleep disorders, has a well-established link with hypertension [2]. While sympathetic activation from intermittent hypoxemia has been recognized as the primary mechanism in obstructive sleep apnea syndrome (OSAS)-related hypertension, emerging evidence suggests additional pathways contribute to blood pressure (BP) elevation. Shen et al. investigated renalase - a kidney-derived flavin-dependent amine oxidase regulating BP through catecholamine degradation - in Chinese Han patients with OSAS. In individuals without OSA, higher renalase concentrations were associated with higher BP, whereas in those with severe OSA, renalase showed an inverse relationship with BP. In OSA, intermittent hypoxia and the resulting oxidative stress activate hypoxia-inducible factor 1α(HIF-1α), which upregulates renalase expression. Elevated renalase appears to function as a compensatory, cytoprotective response - reducing oxidative stress and catecholamine excess - and consequently shows an inverse association with BP in severe OSA. In contrast, in non-OSA individuals where hypoxic stress is absent, renalase levels primarily reflect physiological BP regulation and therefore correlate positively with systolic BP (SBP) and diastolic BP (DBP). Additionally, the renalase gene single nucleotide polymorphism (SNP) rs2296545 polymorphism altered protein function and increased hypertension susceptibility, suggesting the renalase pathway plays a substantial role in BP regulation, particularly in severe OSAS [3].

Beyond resting BP, the relationship between OSAS and exercise-related BP responses has also drawn attention. Exaggerated BP response to exercise has been associated with increased hypertension risk [4], yet few studies have examined relationships between sleep-related breathing disorders (SRBD) and post-exercise BP elevation. A cross-sectional study of 928 transportation workers showed that higher oxygen desaturation index (ODI) correlated with greater exercise-induced BP elevation, suggesting a link between SRBD and abnormal BP recovery [5]. In this study, exercise electrocardiography (ECG) was performed using a treadmill test with the Bruce protocol. The criteria for test termination were as follows: appearance of new symptoms, new significant ECG findings, exercise-induced BP abnormalities (SBP ≥ 250 mmHg or a decrease of ≥10 mmHg), or achievement of the target heart rate (THR). The THR was calculated as (220–age) × 0.85. When exercise-induced BP elevation was defined as an increase in SBP of ≥60 mmHg after the onset of exercise, the 3% ODI level was positively correlated with exercise-induced BP elevation. The risk of exaggerated BP elevation was approximately three-fold higher in participants with a 3% ODI ≥ 15 compared to those with a 3% ODI < 5. On the other hand, a randomized controlled trial (RCT) involving obese adults with moderate-to-severe OSA showed that a 12-week high-intensity interval training program led to significant reductions in peak SBP during exercise, along with improvements in nocturnal BP and apnea-hypopnea index (AHI) [6]. Therefore, although caution is warranted regarding excessive BP elevation during exercise, physical activity remains an important non-pharmacological intervention for obese patients with OSA.

Beyond the diseases themselves, their symptoms are also associated with hypertension risk. A follow-up study of 34,727 subjects from the French CONSTANCES cohort demonstrated that self-reported snoring and excessive daytime sleepiness were associated with increased hypertension risk. Habitual snoring showed modestly increased hazard for developing treated hypertension (hazard ratio [HR], 1.17; 95% confidence interval [CI], 1.03–1.32), whereas excessive daytime sleepiness showed more substantial risk elevation (HR 1.42; 95% CI 1.24–1.62), with clear dose–response associations [7].

Regarding SAS treatment, several noteworthy studies have reported BP-lowering effects of various treatments. The SURMOUNT-OSA trials evaluated tirzepatide, a long-acting dual glucose-dependent insulinotropic polypeptide (GIP) and glucagon-like peptide-1 (GLP-1) receptor agonist, in obese OSAS patient. Tirzepatide reduced AHI[6] by −20.0 and −23.8 events/hour in patients not on positive airway pressure (PAP) therapy and those on PAP therapy, respectively, with concurrent SBP reductions of −7.6 and −3.7 mmHg, highlighting marked benefits in OSAS patients with obesity [8]. The potential mechanism by which tirzepatide reduces BP in patients with OSAS is presumably attributable to substantial reductions in body weight, which in turn may lead to decreased sympathetic activation, improved endothelial function, and reduced systemic inflammation. A significant reduction in high-sensitivity C-reactive protein also supports this hypothesis.

While continuous positive airway pressure (CPAP) remains first-line OSAS treatment, mandibular advancement device (MAD) therapy represents a viable alternative [9, 10]. Meta-analyses have reported comparable antihypertensive effects between MAD and CPAP [11], though many previous studies had limitations including small sample sizes and exclusion of severe OSAS patients. Ou et al. conducted a randomized noninferiority trial comparing MAD versus CPAP effectiveness in reducing 24-h ambulatory BP in 220 patients with moderate to severe OSAS, concomitant hypertension, and elevated CVD risk. After 6 months, MAD was noninferior to CPAP for reducing 24-h mean arterial BP. Both treatments improved daytime sleepiness similarly, with no significant between-group differences in cardiovascular biomarkers [12].

For the antihypertensive effects of nasal surgery for obstruction (NSO) and CPAP therapy in patients with OSAS, it exhibits considerable inter-individual variation unexplained by AHI alone. Messineo and colleagues studied 168 OSAS patients undergoing NSO or CPAP treatment, identifying novel cardiovascular biomarkers of treatment response. Delta heart rate (ΔHR), quantifying heart rate fluctuation magnitude during apneic and hypopneic events, served as a robust predictor of BP improvement. Patients with higher ΔHR values experienced more substantial BP reductions than those with moderate values, with adjusted 24-h SBP difference of 5.8 mmHg (95% CI 1.0-10.5). Additionally, BP improvements were partially mediated through reductions in hypoxic burden (HB) - the cumulative measure of oxygen desaturation depth multiplied by duration across respiratory events during sleep. Changes in HB showed independent association with 24-h SBP reduction of 4.2 mmHg (95% CI 0.9–7.5) per two standard deviation (SD) change. Patients experiencing substantial HB reductions achieved 6.5 mmHg (95% CI 2.5–10.4) greater SBP decrease compared to those with minimal HB changes [13] (Table 1).

**Table 1:** Key points from studies on sleep and blood pressure

Study	Key Points
Renalase and OSAS [3]	Serum renalase levels were elevated in severe OSAS patients and showed an inverse correlation with BP among OSAS subjects. The rs2296545 SNP altered renalase function, increasing susceptibility to hypertension. →Potential role of the renalase pathway in BP regulation, particularly in severe OSAS.
Exercise BP response and SRBD [5]	A higher ODI was associated with greater post-exercise BP elevation, linking sleep-related breathing disorders to abnormal BP recovery and heightened hypertension risk.
SRBD symptoms and hypertension risk [7]	Self-reported snoring and excessive daytime sleepiness were both linked to increased risk of developing hypertension with a stronger dose–response association.
Effects of tirzepatide on BP in obese OSAS [8]	In obese OSAS patients, tirzepatide reduced AHI by 20–24 events/hour and SBP by 3.7–7.6 mmHg, demonstrating significant antihypertensive effects of tirzepatide.
MAD vs. CPAP (Meta-analysis) [12]	In patients with moderate-to-severe OSAS and hypertension, MAD therapy was noninferior to CPAP for 24-h mean arterial BP reduction, with comparable improvements in daytime sleepiness.
ΔHR and hypoxic burden [13]	BP reduction after nasal surgery or CPAP was predicted by higher ΔHR and reductions in HB; those with greater ΔHR and larger HB decreases achieved ~5–7 mmHg greater SBP reduction.

*OSAS* obstructive sleep apnea syndrome, *BP* blood pressure, *SRBD* sleep-related breathing disorders, *ODI* oxygen desaturation index, *AHI* apnea-hypopnea index, *MAD* mandibular advancement device, *CPAC* continuous positive airway pressure, Δ*HR* Delta heart rate, *HB* hypoxic burden, *SBP* systolic blood pressure,

## Air pollution and blood pressure

Environmental pollution has been identified as a hypertension risk factor [14]. World Health Organization (WHO) data indicates approximately 2.1 billion individuals worldwide rely on traditional cooking methods involving open fires or inefficient stoves burning kerosene, biomass materials, and coal, resulting in significant household air pollution exposure [15]. The China Health and Retirement Longitudinal Study (CHARLS) investigated relationships between fuel type and BP outcomes among 3,051 middle-aged and older male participants with baseline prehypertension. Clean fuel usage for heating was associated with improved prehypertension regression compared to solid fuel use (HR 1.28; 95% CI 1.08–1.53). Participants utilizing clean fuels for both heating and cooking showed enhanced prehypertension regression (HR 1.32; 95% CI 1.09–1.60). Consistent clean fuel users for heating exhibited higher likelihood of BP normalization (HR 1.36; 95% CI 1.06–1.73) and demonstrated 2.45 mmHg lower SBP values compared to persistent solid fuel users [16].

Fine particulate matter (PM2.5), a primary air pollution component, has also been linked to elevated BP and increased CVD risk [17]. Brugge and colleagues conducted a randomized crossover trial examining high-efficiency particulate arrestance (HEPA) filtration versus sham filtration effects on BP. In-home HEPA air purifiers produced clinically meaningful SBP reductions, even in relatively low PM2.5 environments. Intervention efficacy was moderated by baseline SBP levels. Participants with elevated SBP (≥120 mmHg) experienced significant 2.8 mmHg mean reduction following HEPA filtration versus 0.2 mmHg increase with sham filtration, yielding significant net 3.0 mmHg decrease by HEPA filtration. No significant benefits were observed for DBP or participants with normal SBP (<120 mmHg). These findings highlight the role of air quality improvement in BP control [18].

## Dietary factors and blood pressure

### Sodium and potassium

Dietary sodium and potassium intake are central modifiable factors in hypertension pathogenesis. Three studies have reported potential new mechanisms linking sodium and potassium to BP that may contribute to future BP management and prevention strategies [19–21].

Yakoub et al. investigated relationships between high salt intake-aggravated immune responses and hypertensive vascular disease development. Apolipoprotein E-deficient mice received transient high salt (HS) exposure (1% NaCl) for 2 weeks followed by washout, then subsequent angiotensin II (Ang II) infusion to induce abdominal aortic aneurysms/dissections and inflammation. They demonstrated that transient HS exposure in mice induced T-cell infiltration into the aorta, which, when combined with Ang II infusion, markedly increased aneurysm and atherosclerosis risk despite no BP difference. The authors proposed a “two-hit model” for hypertensive vascular disease, in which HS primes vascular immunity, enhancing susceptibility to hypertensive injury [19].

Potassium deficiency elevates BP and salt sensitivity. A potassium-dependent signaling pathway in the renal distal convoluted tubule, termed the “potassium switch,” has been implicated in coupling extracellular potassium sensing to thiazide-sensitive NaCl cotransporter (NCC) activation and subsequent NaCl retention [22]. However, whether this pathway causally influences BP remained unclear. Welling et al. addressed this using a genetic approach in mice. In control mice, decreasing plasma potassium concentrations progressively elevated BP and enhanced sensitivity to dietary NaCl and hydrochlorothiazide, coinciding with increased NCC phosphorylation and urinary sodium retention. In contrast, mice with constitutively active SPS1-related proline/alanine-rich kinase (CA-SPAK)—a downstream kinase in the potassium switch pathway—exhibited persistently elevated BP resistant to plasma potassium modulation. These CA-SPAK mice demonstrated salt and diuretic sensitivity across all potassium levels, with sustained NCC hyperactivation and enhanced sodium retention regardless of potassium status [20]. These findings provide causal evidence that potassium deficiency activates the potassium switch pathway, driving NCC-mediated sodium retention, BP elevation, and salt sensitivity.

In salt sensitivity of BP, genetic factors play important roles. Glycosaminoglycans (GAGs), extracellular matrix components involved in salt retention and regulation, are crucial for salinity tolerance [23]. However, relationships between GAG-related genes and BP regulation remained unclear. A genome-wide interaction study using the EPIC-Norfolk cohort (approximately 20,000 participants) analyzed associations between SNPs in GAG genes, urinary sodium excretion, and BP. Validation was performed using UK Biobank (*n* = 414,132) and multiethnic HELIUS cohort (Healthy Life in an Urban Setting; *n* = 2239) data. The study identified significant gene-sodium interactions in NDST3 gene (rs2892799) and HS3ST5 gene (rs9654628). Individuals carrying the C allele of rs2892799 exhibited elevated mean arterial pressure under high sodium conditions, whereas those with T allele showed no such sodium-dependent BP response. The rs9654628 variant in HS3ST5 demonstrated significant interaction with sodium intake on SBP. These findings were validated in the multiethnic HELIUS cohort. A dietary sodium loading crossover study enrolling a subset of HELIUS participants revealed that urinary GAG expression differed by rs2892799 genotype. Individuals with stable BP after sodium loading showed increased urinary expression of N-sulfated heparan sulfate epitope D0S0, whereas those with sodium-induced BP elevation exhibited opposite patterns. These results suggest salt sensitivity is influenced by genetic variants in NDST3 and HS3ST5, and these genes modulate urinary GAG composition [21]. Differential GAG profiles may mechanistically link genetic variation to salt-sensitive hypertension phenotypes.

From a clinical perspective, several interesting findings have emerged regarding sodium restriction and BP reduction. Sodium restriction is essential even for patients receiving antihypertensive medications. However, existing data regarding the interaction between pharmacological therapy and the BP-lowering effects of sodium reduction have yielded conflicting results. A recent meta-analysis encompassing 35 RCTs including 2885 participants provided important insights. The analysis revealed that each 100 mmol decrease in 24-h urinary sodium excretion was associated with reductions of 6.81 mmHg in SBP (95% CI 4.96–8.66), 3.85 mmHg in DBP (95% CI 2.26–5.43), and 4.83 mmHg in mean arterial pressure (95% CI 3.22–6.44). Notably, BP response magnitude to sodium restriction demonstrated significant heterogeneity depending on antihypertensive drug class employed. Patients receiving β- blockers, renin-angiotensin-aldosterone system (RAS) inhibitors, or combination therapy exhibited more pronounced BP reductions compared to those treated with calcium channel blocker (CCB) or diuretic monotherapy. The reason why sodium restriction produced more pronounced BP-lowering effects with β-blockers and RAS inhibitors compared with diuretics and CCBs remains unclear. However, for diuretics, only two RCTs were included in this meta-analysis, which may have resulted in insufficient statistical power. For CCBs, natriuretic effects are enhanced in individuals with higher sodium intake [24]; therefore, additional sodium restriction in patients whose sodium balance has already been affected by these agents may have yielded only modest further reductions in BP. Howeber, these explanations remain speculative, and further investigation is warranted to elucidate the mechanisms underlying the BP-lowering effects of sodium restriction and the differences across drug classes. Nonetheless, BP response to sodium restriction remained consistent across various patient characteristics. No significant differences were identified when participants were stratified by age, baseline urinary sodium excretion levels, initial BP values, or study duration [25]. These findings revealed the importance of maintaining sodium restriction as complementary strategy to pharmacological therapy.

Moreover, multiple noteworthy reports have examined the effects of sodium reduction at the population level. Recent evidence suggests community-wide interventions delivered through institutional settings—including educational facilities, workplaces, and healthcare institutions—can be implemented efficiently and may achieve BP reductions comparable to individualized dietary counseling approaches. Nevertheless, studies evaluating sodium restriction strategy effectiveness at population levels remained scarce. A comprehensive meta-analysis undertaken as part of evidence synthesis for Japanese Society of Hypertension Guidelines 2025 development [26], incorporating 36 RCTs with combined enrollment of 66,803 participants, evaluated population-based salt reduction intervention impact on BP outcomes. The analysis demonstrated that population-based sodium restriction programs significantly reduced office SBP compared to standard care, with mean difference of −2.64 mmHg (95% CI − 3.48 to −1.80). While significant benefits were observed in adult populations, trials exclusively involving pediatric participants failed to demonstrate clear efficacy [27]. Notably, enhanced therapeutic responses were observed among hypertensive individuals and Asian populations. The study also confirmed significant reductions in dietary sodium consumption across intervention groups [27]. These findings provide robust evidence supporting population-level salt reduction strategy effectiveness as viable public health approach for BP control, particularly when targeted toward high-risk populations and implemented in occupational settings.

While recommendations for pharmacological intervention in prehypertension vary across hypertension guidelines, comparative cost-effectiveness of antihypertensive medication versus dietary sodium reduction strategies in this patient population remained unclear. A recent cost-effectiveness analysis in the Chinese population employed a Markov cohort model to simulate lifetime cardiovascular disease events, healthcare costs, and quality-adjusted life years (QALYs). The study compared three intervention strategies: salt substitution alone, antihypertensive drug therapy alone, and combination of both approaches. The analysis examined outcomes in the general prehypertensive population and stratified by CVD risk level and intervention initiation age (40, 50, 60, and 70 years)[28]. For the overall prehypertensive population, salt substitution initiated at age 40 emerged as the only cost-effective single intervention, demonstrating incremental cost-effectiveness ratio of $6,413.62 per QALY gained. Among high-CVD-risk individuals, combination strategy starting at age 40 proved most cost-effective, with incremental cost-effectiveness ratio of $2,913.30 per QALY. Notably, earlier intervention initiation consistently yielded greater CVD risk reduction and more favorable cost-effectiveness profiles [28]. For instance, combined intervention beginning at age 40 reduced CVD events by 5.3% with incremental cost-effectiveness ratio of $2,913.30 per QALY, compared to 4.9% reduction and incremental cost-effectiveness ratio of $32,635.33 per QALY when initiated at age 70 [28]. These findings suggest that in the Chinese context, substituting regular salt with potassium-enriched alternatives represents a more cost-effective approach than antihypertensive medication for prehypertensive adults over 40 years. Furthermore, data indicate that initiating interventions at younger ages in prehypertensive individuals may yield substantial additional cost savings and health benefits.

From technological perspective, innovative reports have emerged regarding sodium restriction strategies. Recently, electrical taste stimulation, which modulates food perception through electrical activation of the tongue and surrounding tissues, has emerged as an innovative strategy [29]. Studies have demonstrated that electrically-enhanced utensils can successfully modulate saltiness and umami perception in various foods [30, 31]. Despite practical advantages, their effectiveness is limited to periods of direct contact between utensil, food, and tongue. This limitation necessitates developing alternative electrical stimulation methods suitable for solid foods requiring mastication. Transcutaneous electrical stimulation (TES) has gained recognition as a promising technique providing sustained taste modification. Research has shown that anodal TES (aTES), achieved by positioning electrode gels on the anterior mandibular region (anodal site) and posterior neck area (cathodal site), significantly enhances salt perception in sodium chloride solutions. Compared to direct lingual electrical stimulation, aTES offers the distinct advantage of maintaining taste enhancement throughout both mastication and swallowing phases [32]. Funamizu and colleagues studied 27 female Japanese university students in a sensory evaluation employing Quantitative Descriptive Profile methodology. They examined aTES-induced modifications in taste characteristics across six processed food products: cold potato potage, chicken broth soup, rice porridge with pickled plums, Chinese pork stir fry, stir-fried pork and radish, and fried dumplings. aTES application resulted in statistically significant enhancements in both salt perception and overall taste intensity across all six food products tested. Further sensory analysis of 32 processed food items revealed selective modulation of specific flavor attributes. For example, significant aTES-associated increases were observed in umami characteristics of dumplings, while reductions were noted in several flavor components such as chicken broth notes and sweetness in potato soup [33]. While aTES technology effectiveness requires further validation across diverse food categories varying in cultural origin, nutritional composition, and taste profiles, these findings highlight its potential as an important tool for future sodium reduction strategies. The ability to enhance saltiness perception while maintaining or improving palatability in certain foods suggests electrical taste modulation may offer a viable complement to traditional dietary sodium restriction approaches.

In concluding this section on sodium, potassium, and BP, it is important to acknowledge the statement on sodium-to-potassium ratio released by the Japanese Society of Hypertension in 2024 [34]. The Society recommended collecting casual urine samples at various times across at least four days per week for measurement. The Society proposed an average urinary Na/K ratio of 2 as the optimal target value and suggested an average ratio of 4 as a feasible interim target [34]. For comprehensive information regarding this topic, readers are referred to the statement [34] (Table 2).

**Table 2:** Sodium, potassium, and blood pressure

Study	Key Points
**Mechanistic Perspective**	
High-salt immune activation model [19]	Transient high salt exposure primes vascular immunity through T-cell infiltration, creating a “two-hit model” where subsequent angiotensin II exposure markedly increases aneurysm and atherosclerosis risk independent of BP changes.
Potassium switch pathway [20]	Potassium deficiency activates the “potassium switch” pathway in renal distal convoluted tubule, driving NCC-mediated sodium retention and BP elevation. Genetic activation of SPAK kinase causes persistent hypertension resistant to potassium modulation.
GAG gene and salt sensitivity [21]	Genetic variants in NDST3 (rs2892799) and HS3ST5 (rs9654628) genes modulate salt sensitivity through differential urinary GAG expression. Carriers of specific alleles exhibit sodium-dependent BP responses linked to altered GAG profiles
**Clinical Perspective**	
Sodium restriction and antihypertensive drugs (Meta-analysis) [25]	Each 100 mmol reduction in urinary sodium excretion lowered SBP by 6.81 mmHg, with stronger effects among patients on β-blockers or RAS inhibitors compared to calcium channel blockers or diuretics.
**Public Health Perspective**	
Effects of Population-based sodium reduction on BP (Meta-analysis) [27]	Population-based sodium restriction programs reduced office SBP by −2.64 mmHg, showing significant benefits in adults, particularly among hypertensive and Asian populations.
Cost-effectiveness analysis of salt substitution vs. drugs in prehypertension [28]	Salt substitution was more cost-effective than antihypertensive medication in prehypertensive Chinese adults over 40 years. Earlier intervention initiation yields greater CVD risk reduction and better cost-effectiveness.
**Technological Perspective**	
Anodal transcutaneous electrical stimulation [33]	aTES significantly enhances salt perception and overall taste intensity across processed foods, offering a novel technological approach to facilitate dietary sodium reduction while maintaining palatability.

Representative studies from the review are categorized into mechanistic, clinical, and public health perspectives. Each study’s key point is summarized in 1–2 concise sentences

*BP* blood pressure, *NCC* thiazide-sensitive NaCl cotransporter, *GAG* Glycosaminoglycan, *SBP* systolic blood pressure, *RAS* renin-angiotensin-aldosterone system, *CVD* cardiovascular disease, *aTES* Anodal transcutaneous electrical stimulation

## Dietary fiber and blood pressure

Dietary fiber is recommended in multiple guidelines, including the Japanese Society of Hypertension 2025 [26]. However, in dialysis patients, intake of vegetables, fruits, and seaweed—primary dietary fiber sources—is often restricted to prevent hyperkalemia. Nagasawa et al. conducted a single-arm study in Japanese hemodialysis patients examining associations between gut microbiota and CVD risk factors, including BP and uremic toxin indoxyl sulfate. Participants consumed fruit granola as breakfast replacement for 8 weeks. The intervention resulted in reductions in SBP and DBP, estimated salt intake, and serum indoxyl sulfate concentrations. Additionally, stool consistency assessed by Bristol Stool Form Scale improved. Gut microbiota analysis revealed increased α-diversity along with enrichment of Blautia and Neglecta species. Lactic acid-producing and ethanol-producing bacteria significantly increased, while indole-producing bacteria significantly decreased [35]. These findings suggest fruit granola consumption may serve as a useful approach for sodium reduction, dietary fiber supplementation, and gut microbiota modulation in dialysis patients.

However, evidence regarding optimal fiber intake recommendations remains limited. One review suggested that minimum daily dietary fiber intake for adults with hypertension should exceed 28 g/day for women and 38 g/day for men. Each additional 5 g/day of fiber was estimated to reduce SBP by 2.8 mmHg and DBP by 2.1 mmHg [36]. Such intake levels may support healthy gut microbiota composition and enhance production of short-chain fatty acids, gut microbiota-derived metabolites with BP-lowering properties.

## Other dietary factors and blood pressure

Ultraprocessed foods (UPF), defined as ready-to-eat products formulated from processed substances derived or refined from whole foods and generally containing added flavoring agents, coloring agents, and various cosmetic additives, have raised concerns regarding their association with various disease risks. A study utilizing data from 5957 participants in the REGARDS (Reasons for Geographic and Racial Disparities in Stroke) cohort examined longitudinal relationships between UPF intake and incident hypertension, while exploring UPF consumption’s contribution to racial disparities in hypertension risk. Over an average follow-up period of 10 years, results demonstrated a positive linear relationship between UPF consumption level and hypertension risk. The findings indicated that racial differences in UPF intake patterns contribute to disparities in hypertension risk, with Black adults exhibiting higher consumption levels associated with elevated hypertension risk [37].

While associations between antioxidants and BP have been previously reported [38], a Chinese cross-sectional study examined two comprehensive dietary antioxidant measures rather than individual antioxidants. The study assessed Dietary Total Antioxidant Capacity (DTAC), a tool evaluating overall antioxidant potential of daily diets, and Dietary Antioxidant Quality Score (DAQS), an index of combined intake of key dietary antioxidant nutrients including vitamins C, A, and E, selenium, and zinc. Results showed a significant non-linear relationship between DTAC and lower BP values and hypertension prevalence, while DAQS demonstrated a linear relationship with these outcomes [39]. The differential relationship patterns—non-linear for DTAC versus linear for DAQS—likely reflect distinct mechanisms. DTAC captures synergistic effects among various dietary antioxidants with potential threshold effects, while DAQS focuses on specific nutrients with established biological roles, explaining its linear dose-response. This highlights the importance of evaluating dietary patterns holistically rather than focusing on individual nutrients. Dietary habits recommended for hypertension management are also described in the latest Japanese Society of Hypertension Guidelines 2025 (JSH 2025) [26]. Specifically, these include sodium restriction to less than 6 g/day; active potassium intake (≥3000 mg/day for adult men, ≥2,600 mg/day for adult women), achieved through consumption of vegetables (350 g/day), fruits (200 g/day), and low-fat milk and dairy products (however, potassium intake should be restricted in patients with renal impairment or other conditions requiring potassium limitation); adequate intake of calcium, magnesium, dietary fiber, and unsaturated fatty acids; and alcohol cessation (≤20–30 mL/day of ethanol for men, ≤10–20 mL/day for women). Regarding dietary patterns, the benefits of the DASH diet and Mediterranean diet are highlighted. Additionally, fish-based meals rather than meat-based meals, whole grains, legumes, and unsalted nuts as snacks are recommended. Regarding meal timing, a systematic review has reported that late eating patterns—such as having lunch after 1:00 PM or consuming the largest proportion of daily energy intake in the late evening—are associated with an increased risk of hypertension [40]. Although the evidence has limitations due to heterogeneity in the definitions and measurement methods of meal timing across studies, consuming meals earlier in the day may better align with the body’s natural circadian rhythm and potentially improve cardiometabolic health [41]. Therefore, it may be advisable to avoid heavy meals late at night and to consume meals earlier in the day whenever possible.

Recent studies from diverse countries in 2024 have strengthened the evidence linking lifestyle factors to BP regulation, though much remains unknown about the underlying mechanisms. Lifestyle modification remains the cornerstone of non-pharmacological BP management, regardless of advances in pharmacotherapy or medical technology. Continued efforts to elucidate causal mechanisms, refine individualized strategies, and inform public health policies are warranted.
